# Positive mental health and COVID-19 pandemic in Slovenia

**DOI:** 10.1093/eurpub/ckac131.474

**Published:** 2022-10-25

**Authors:** M Vinko, P Mikolič, S Roškar, H Jeriček Klanšček

**Affiliations:** Centre for Health Research and Development, National Institute of Public Health, Ljubljana, Slovenia

## Abstract

**Background:**

The COVID-19 pandemic is having a major impact on mental health. In contrast to studies on mental disorders, our study contributes to the under-researched area of the impact of the pandemic on positive mental health. We compare the prevalence of positive mental health before and during the pandemic and identify factors associated with flourishing and languishing mental health during the pandemic.

**Methods:**

We used data from two nationally representative cross-sectional surveys of Slovenian adults conducted in 2019 and 2021. Outcome included positive mental health (Mental Health Continuum - Short Form). Cross-sectional prevalence estimates were calculated and logistic regression assessed associations between positive mental health and the COVID-19 specific and other health-related factors.

**Results:**

In 2021, 38.6% (95% CI, 37.0%-40.2%) had flourishing mental health, compared to 61,5% (95% CI, 60.5%-62.5%) before the pandemic. In contrast, the share of people in languishing mental health during the pandemic (8.0%; 95% CI, 7.1%-8.9%) was nearly two times higher than in 2019 (4,5%; 95% CI, 4.1%-4.9%). Both flourishing and languishing were significantly associated with changes in family relations, social interactions and dietary habits, resilience and COVID-19 literacy.

**Conclusions:**

Positive mental health in Slovenia worsened drastically during the pandemic compared with before. Results indicate important role of family relationships, social interactions and dietary habits on positive mental health. Both prevention of mental disorders and mental health promotion need to be considered in order to address the full range of public mental health needs, with increased attention to strengthening resilience and health literacy.

**Key messages:**

• We noted an substantial decline in flourishing and rise in languishing mental health during the pandemic.

• Public health efforts need to address the impact of the pandemic on family relations and social interactions.

